# Molecular and spatial heterogeneity of microglia in Rasmussen encephalitis

**DOI:** 10.1186/s40478-022-01472-y

**Published:** 2022-11-21

**Authors:** Jesse J. Westfall, Wesley N. Schwind, Sahibjot Sran, Jason B. Navarro, Jeffrey Leonard, Jonathan A. Pindrik, Christopher R. Pierson, Daniel R. Boué, Daniel C. Koboldt, Adam P. Ostendorf, Richard K. Wilson, Elaine R. Mardis, Katherine E. Miller, Tracy A. Bedrosian

**Affiliations:** 1 The Steve and Cindy Rasmussen Institute for Genomic Medicine, Abigail Wexner Research Institute at Nationwide Children’s Hospital, Research Building 3, 575 Children’s Crossroad, Columbus, OH 43215 USA; 2 Department of Neurosurgery, Nationwide Children’s Hospital, Columbus, OH USA; 3 Department of Neurosurgery, The Ohio State University College of Medicine, Columbus, OH USA; 4 Department of Pathology, The Ohio State University College of Medicine, Columbus, OH USA; 5 Department of Pathology and Laboratory Medicine, Nationwide Children’s Hospital, Columbus, OH USA; 6 Department of Biomedical Education and Anatomy, Division of Anatomy, The Ohio State University College of Medicine, Columbus, OH USA; 7 Department of Pediatrics, The Ohio State University College of Medicine, 700 Children’s Dr., Columbus, Oh 43205 USA; 8 Division of Child Neurology, Nationwide Children’s Hospital, Columbus, OH USA

**Keywords:** Encephalitis, Microglial nodules, Epilepsy, Inflammation, Spatial proteomics, Single nucleus RNA-seq, Cytokine signaling

## Abstract

Rasmussen encephalitis (RE) is a rare childhood neurological disease characterized by progressive unilateral loss of function, hemispheric atrophy and drug-resistant epilepsy. Affected brain tissue shows signs of infiltrating cytotoxic T-cells, microglial activation, and neuronal death, implicating an inflammatory disease process. Recent studies have identified molecular correlates of inflammation in RE, but cell-type-specific mechanisms remain unclear. We used single-nucleus RNA-sequencing (snRNA-seq) to assess gene expression across multiple cell types in brain tissue resected from two children with RE. We found transcriptionally distinct microglial populations enriched in RE compared to two age-matched individuals with unaffected brain tissue and two individuals with Type I focal cortical dysplasia (FCD). Specifically, microglia in RE tissues demonstrated increased expression of genes associated with cytokine signaling, interferon-mediated pathways, and T-cell activation. We extended these findings using spatial proteomic analysis of tissue from four surgical resections to examine expression profiles of microglia within their pathological context. Microglia that were spatially aggregated into nodules had increased expression of dynamic immune regulatory markers (PD-L1, CD14, CD11c), T-cell activation markers (CD40, CD80) and were physically located near distinct CD4+ and CD8+ lymphocyte populations. These findings help elucidate the complex immune microenvironment of RE.

**Supplementary Information:**

The online version contains supplementary material available at 10.1186/s40478-022-01472-y.

## Introduction

Rasmussen encephalitis (RE) is a rare, progressive neurological disease characterized by intractable seizures and unilateral neurological deficits with atrophy of the contralateral cerebral hemisphere [62]. The disorder mainly affects children, with a median onset around 6 years of age, and an estimated incidence of 1.7 to 2.4 cases per 10 million children [10]. The etiology of RE is unknown, but the evidence points to an immunopathological mechanism, particularly involving T-cell cytotoxicity and microglial activation. On histopathologic examination, brain tissue from RE patients typically shows signs of cortical inflammation, infiltrating cytotoxic T-cells, microglial nodules, neuronal loss, and gliosis in the affected hemisphere [48]. As the disease progresses, cortical cavitation and profound degeneration are observed [44].

RE remains a disease with poorly understood etiology [17, 58]. For a decade, studies have implicated microglia and their interaction with infiltrating T-cells in RE disease progression and neurodegeneration [42, 43, 62]. The quantification of these intercellular mechanisms, and their contributions to RE pathogenesis, has remained elusive. Omics analysis at tissue resolution and traditional low-plex immunohistochemistry have made incremental advances to elucidate the complex network of cytokine-mediated signaling driving the inflammatory immune response, but much remains to be determined. Diagnostic challenges further obscure the origin of RE pathogenicity, particularly in early-stage RE. Lacking distinct disease biomarkers, diagnosis relies on clinical, morphological and electrophysiological criteria [41]. Atypical presentation can elude clinical diagnosis and there are no specific EEG abnormalities that can distinguish RE from other focal epilepsy [41, 58]. Neuroimaging is used to identify the unilateral inflammation and atrophy that characterizes RE but is often negative in early stages [62]. These shortcomings highlight the need for high-resolution, multiomic analyses.

Several studies have characterized aspects of innate and adaptive immune activity in RE. At early disease stages, there is an upregulation of innate immune response including MHC-class-1 antigen presentation and interferon signaling [60]. The formation of small microglial nodules expressing Toll-like receptor 7 precedes infiltration of lymphocytes to the brain. As the disease progresses, gene expression of chemokines responsible for T-cell attraction, such as *CCL5*, *CXCL9*, and *CXCL20*, are more abundantly expressed in RE patient brains, as well as inflammatory gene expression including *IL-1β* and *IFNγ* [43, 60]. Infiltration and clonal T-cell expansion occurs in the brains of RE patients and CD8 + cytotoxic T-cells localize to the microglial nodules [1, 52, 53]. These T-cells can be observed closely apposed to neurons, where it is believed they may recognize a specific epitope that is yet to be identified [53].

These findings suggest that the microglial response is a key component of RE pathogenesis, but studies so far have relied on bulk tissue gene expression assays that do not allow for cell-type resolution. Here we applied single-nucleus RNA-sequencing (snRNA-seq) to identify cell-type-specific gene expression changes in RE, with a particular focus on microglia. We extended these findings by applying spatial proteomic assays to study expression of microglia within their pathological context. Our results help elucidate the complex immune microenvironment of RE.

## Materials and methods

### Patient cohort

Samples RE1, RE2, RE3, FCD1 and FCD2 were collected from patients diagnosed with either Rasmussen encephalitis (RE) or Type I focal cortical dysplasia (FCD) and enrolled in an IRB-approved research study at Nationwide Children’s Hospital. Samples RE2 and RE3 originated from the same patient, but from different surgeries three years apart over the course of disease progression. Formalin-fixed, paraffin embedded (FFPE) tissue samples for RE4 were obtained from the Cooperative Human Tissue Network, a resource supported by the National Cancer Institute. Samples CTRL1 and CTRL2 were collected from unaffected donors at autopsy courtesy of the NIH Neurobiobank. See Table 1 for details.

**Table 1:** Sample information

Sample ID	Patient Age at Surgery	Sex	Diagnosis	Sample origin	Brain region studied	Frozen tissue for snRNA-seq	FFPE slides for spatial proteomics
RE1	0–5	F	RE	Left-sided functional hemispherectomy	Anterior temporal lobe	X	X
RE2	6–10	F	RE	Left anterior temporal lobectomy	Anterior temporal lobe		X
RE3	6–10	F	RE	Left peri-insular functional hemispherectomy	Posterior temporal lobe	X	X
RE4	6–10	F	RE	Right anatomic hemispherectomy	Temporal lobe		X
FCD1	11–15	F	FCD Type I	Excision of left superior parietal and occipital lobe	Superior parietal and occipital lobe	X	
FCD2	11–15	F	FCD Type I	Resection of right inferior parietal epileptic zone	Middle frontal gyrus	X	
CTRL1	0–5	F	Deceased (Lymphocytic Myocarditis)	Autopsy – temporal cortex (Brodmann area 22)	Temporal cortex	X	
CTRL2	0–5	F	Deceased (Respiratory Failure)	Autopsy – temporal cortex (Brodmann area 22)	Temporal cortex	X	

### Single-nucleus RNA-sequencing

#### Nuclei isolation

Nuclei were isolated from frozen brain tissue as previously described [29]. Briefly, ~ 20 mg of tissue was mechanically homogenized using a glass Dounce homogenizer. Nuclei were washed and stained with Hoechst 33,342 dye (Thermo Fisher Scientific #62,249), filtered through a 30 µm mesh filter, and resuspended in PBS. FACS analysis was performed using an Influx Cell Sorter (BD Biosciences). Cellular debris were excluded using forward and side scatter area parameters, nuclei were gated on Hoechst-positive signal, and then aggregates were excluded using trigger pulse width. Purified Hoechst-positive nuclei were sorted directly into 10x Genomics reaction buffer and processed according to the manufacturer protocol for Chromium Next GEM Single-Cell 3’-Reagent Kit v.3.1. Final libraries were sequenced either on an Illumina NovaSeq 6000 or HiSeq 4000 instrument to generate paired-end sequencing data.

#### Data pre-processing

Generation of FASTQ files, read alignment to the GRCh38 transcriptome and quantification of cell feature counts was performed using 10x Genomics Cell Ranger v.6.0 following default parameters, except for inclusion of intronic transcripts, which are present in nucleic pre-mRNAs.

### Quality filtering, normalization, and integration

Downstream analysis was performed using Seurat v4.0 for R [23]. Briefly, cell-barcode gene expression matrices were filtered to contain genes that were expressed in at least three nuclei and to contain nuclei that express at least 1000 transcripts of 500 unique genes. Nuclei with greater than 5% mitochondrial transcripts were filtered out. Normalization and variance-stabilization of feature-barcode matrices were performed using the sctransform package for R [22]. Nuclear doublets were detected and eliminated from the data via DoubletFinder v2.0 [36]. Feature-barcode matrices were integrated using the IntegrateData function in Seurat.

### Dimensionality reduction, clustering and visualization

Principal component analysis (PCA) was performed using Seurat v.4.0. The first 30 PCs were used to generate a shared nearest neighbor (SSN) graph by calculating the Jaccard Index between every nucleus and its 20 nearest neighbors. The resulting SSN graph was used to perform Louvain clustering [59]. Gene expression profiles were visualized in two-dimensional space using t-distributed stochastic neighbor embedding (tSNE) [61].

### Cell type annotation

Cell types were annotated using the reference-query label transfer method in Seurat v.4.0 using human cortical single-nuclei gene expression data from the Allen Brain Map M1 10x dataset [7]. Briefly, a set of transfer anchors is calculated from the PCA reduction of each dataset. Then, each nucleus in the query dataset is scored on a reference vector of cell type labels, generating a matrix of prediction scores and predicted IDs to be added to the query’s metadata. To confirm predicted cell types and find nuclei from non-resident nuclei, canonical cell type markers were visualized with the Nebulosa v3.14 R package [2].

### Normalized cell type quantification

To correct for sample size when reporting cell type proportions, the number of nuclei in each cell type cluster was normalized to total nuclei in each sample using the following formulas, as previously published [51].

$$Normalization\;{\text{Factor}}\left( {NF} \right) = \frac{{\left( {nuclei\;in\;sample} \right)}}{{\left( {nuclei\;in\;sample\;with\;largest\;number\;of\;nuclei} \right)}}$$

$$normalized\;cell\;number = \frac{{\left( {number\;of\;nuclei\;in\;cell\;type\;clusterina\;sample} \right)}}{{NF}}$$

### Subclustering and differential gene expression

Microglia were subset from the integrated Seurat object and reprocessed using normalization, integration, dimensional reduction, and clustering steps as described above. To discover differentially expressed genes (DEGs) between disease conditions, we used the pseudobulk method of aggregating gene expression across cells of biological replicates, then employed DESeq2 to test for differential expression [33]. Dimensionality reduction, Louvain clustering and visualization were performed using Seurat v.4.0 as described above. Highly expressed genes were discovered on a per-cluster basis using the FindAllMarkers function in Seurat v.4.0 with test.use set to Model-based Analysis of Single-cell Transcriptomics (MAST) [18].

### GO Enrichment and KEGG Pathway analysis

Gene Ontology enRIchment anaLysis and visuaLizAtion tool (GOrilla) was used to analyze DEGs in microglia across disease conditions and across microglia clusters [15]. All genes with transcripts sequenced across the RE, FCD1 and CTRL samples were used as a background list. DEGs were queried for enriched Gene Ontology (GO) biological process annotations (FDR adjusted *p*-value < 0.05). Additionally, microglial DEGs across disease conditions were input into the Kyoto Encyclopedia of Genes and Genomes (KEGG) Pathway Database Mapper Search Tool [25] for pathway analysis and visualization.

### Cell typing statistics

To test significance of microglial cluster association to disease condition, an entropy based metric, the cell type diversity statistic (CTDS) was employed [26]. CTDSs were normally distributed and maintained homogeneity of variance, confirmed by Shapiro–Wilk test and Bartlett’s test respectively, and tested via one-way ANOVA.

### Trajectory analysis

Trajectory analysis was performed via Monocle3 [46]. Microglia were dimensionally reduced via Uniform Manifold Approximation and Projection (UMAP) with Seurat v.4.0. Using UMAP loading scores and Louvain clusters, cells were ordered along computationally generated pseudotime trajectories using the ‘learn_graph’ and ‘order_cells’ functions. Differentially expressed genes across pseudotime were found by spatial autocorrelation (Moran’s I test) using the ‘graph_test’ function.

### Digital spatial proteomics

#### Slide preparation

FFPE tissue sections (5 µm thick) were processed according to the GeoMx® Digital Spatial Profiler (DSP) protocol (NanoString Technologies, Inc.). Briefly, tissue was deparaffinized and rehydrated with CitriSolv (Decon Labs), ethanol and ddH_2_O washes at room temperature. Antigen retrieval was performed in citrate buffer pH 6.0 (Millipore) at ~ 115 °C under high pressure and washed in tris-buffered saline with Tween 20 (TBS-T) (Cell signaling Technology). Tissue was blocked with Buffer W (Nanostring) for 1 h at room temperature. Slides were incubated at 4 °C overnight with antibody mixture including fluorescently tagged antibodies for IBA1-AF532 (Nanostring Technologies, Inc. #121,300,306) and CD45-AF594 (NanoString Technologies, Inc. #121,300,301) and the following oligo-tagged NanoString GeoMx human detection antibody mixtures: Immune Core Profiling, Immune Cell Typing, Immune Activation, and Cell Death (see Supplemental Material for list of detection targets). Slides were washed with TBS-T, fixed with 4% formaldehyde (Invitrogen) for 1 h at room temperature, then re-washed with TBS-T. Nuclei were stained with SYTO 13 (NanoString).

#### GeoMx instrument use and analysis

Slides were scanned in a GeoMx DSP instrument. Geometric regions of interest (ROIs) were selected and segmented for each tissue section, focusing on areas containing microglial nodules and aggregated immune cells. For each ROI, CD45+ segment borders were generated by manually selecting a CD45 fluorescence threshold that visually maximized, based on intensity and cell morphology, inclusion of invading immune cells while excluding microglia. Subsequently, IBA1+ segment borders were generated by manually selecting an IBA1 fluorescence threshold that visually maximized inclusion of microglia while excluding non-cellular area. Oligos from selected ROI segments were cleaved, collected and dried in a 96-well plate. Oligos were hybridized to unique reporter tags and counted with the Nanostring nCounter platform following the manufacturer protocol. The number of selected ROI segments per sample is detailed in Table 2.

**Table 2:** GeoMx sample ROI information

Sample Name	Slide #	# IBA1+ ROI segments	# CD45+ ROI segments
RE1	1	13	10
RE1	2	21	15
RE2	1	14	10
RE2	2	11	7
RE3	1	15	9
RE3	2	11	7
RE4	1	14	10
RE4	2	13	9

Data quality control, normalization and differential expression analysis were performed on NanoString GeoMx Software v2.4 following manufacturer recommendations. Differentially expressed proteins were determined by Mann Whitney U Test with *p*-values corrected by Benjamini–Hochberg procedure (*p* < 0.05). Relative expression of cell type markers and cell activation markers was calculated as follows:

$$Cell\;marker\;\% \;Counts = \frac{{\#\; of\;oligos\;from\;specific\;cell\;type\;marker}}{{total\;\# of\;oligos\;from\;all\;cell\;type\;markers}} \times 100$$

$$Cell\;activation\;\% \;Counts = \frac{{\#\; of\;oligos\;from\;specific\;cell\;activation\;marker}}{{total\;\# of\;oligos\;from\;all\;cell\;activation\;markers}} \times 100$$

### Immunohistochemistry

Formalin-fixed paraffin-embedded tissue sections (5 µm thick) were processed according to the GeoMx Digital Spatial Proteomics protocol (NanoString Technologies, Inc.), as above, with the addition of CD8-AF647 (Novus #NBP2-54595AF647) and the exclusion of GeoMx human detection antibodies. High-resolution full-slide images at 20 × were obtained via the GeoMx DSP.

### Microglial expansion and infiltrating lymphocytes in RE

**Fig. 1:**
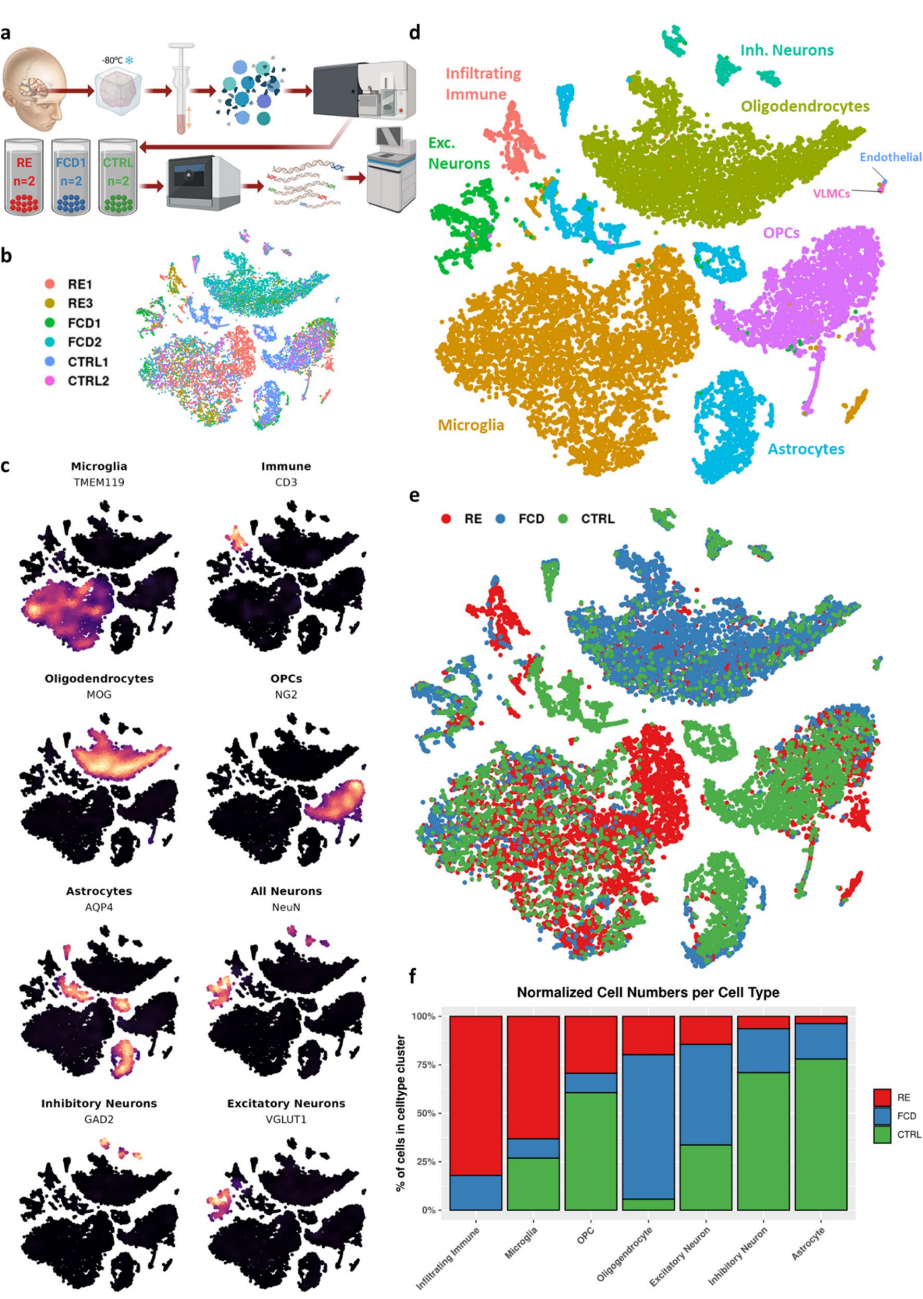
Microglial and lymphocytic cell populations are expanded in Rasmussen encephalitis. **a** Processing of cortical tissue from RE (*N* = 2), FCD (*N* = 2), and unaffected (*N* = 2) individuals for snRNA-seq was performed using 10x Genomics 3’gene expression kit. **b** tSNE plot highlighting patient contribution to each cluster. **c** tSNE plots overlaid with feature density for canonical cell type markers (endothelial and VLMC not shown) **d** tSNE plot colored by cell type, determined using Seurat v.4.0 reference and query workflow (see methods). **e** tSNE showing contribution of diagnosis by cluster. **f** Normalized counts of nuclei per cluster by diagnosis

To determine the cell type composition of RE-affected cortical tissue, we performed snRNA-sequencing of frozen brain tissue from RE patients (*N* = 2) compared to Type I FCD patients (*N* = 2) and unaffected donors (*N* = 2). FCD samples were used as an epilepsy control without any known immunological pathology, and unaffected donors had neither history of epilepsy nor immune disease. We performed unbiased nuclei isolation using mechanical dissociation and fluorescence-activated sorting for Hoechst-positive nuclei. Individual nuclei were barcoded (10x Genomics, Inc.) and cDNA libraries were sequenced (Fig. 1a). After quality control, snRNA-seq yielded 20,182 single-nuclei profiles (nuclei counts: RE = 8,779; FCD = 5,353; CTRL = 6,050) (Additional file 1: Fig. 1a–e). An average of 2,526 genes and 6,127 transcripts per nucleus were detected. We normalized the data, applied dimensional reduction analysis and performed unbiased clustering to identify cell types (Fig. 1b–c; Aditional file 1: Fig. 1f). Nine major cell types were identified and confirmed by expression of canonical cell-type markers (Fig. 1d). Expected brain resident cell types were identified, including neurons, oligodendrocytes, astrocytes, and microglia. Microglia were highly represented in the RE patient samples, with more than twice as many identified after normalizing for sample size compared to FCD or CTRL samples. A population of non-resident, infiltrating immune cells identified with the markers CD3, SKAP1 and CD94 originated mainly from the RE patient samples and was absent from unaffected tissue. (Fig. 1e–f; Additional file 1: Fig. 1 g-h).

### Microglial gene expression in RE

**Fig. 2:**
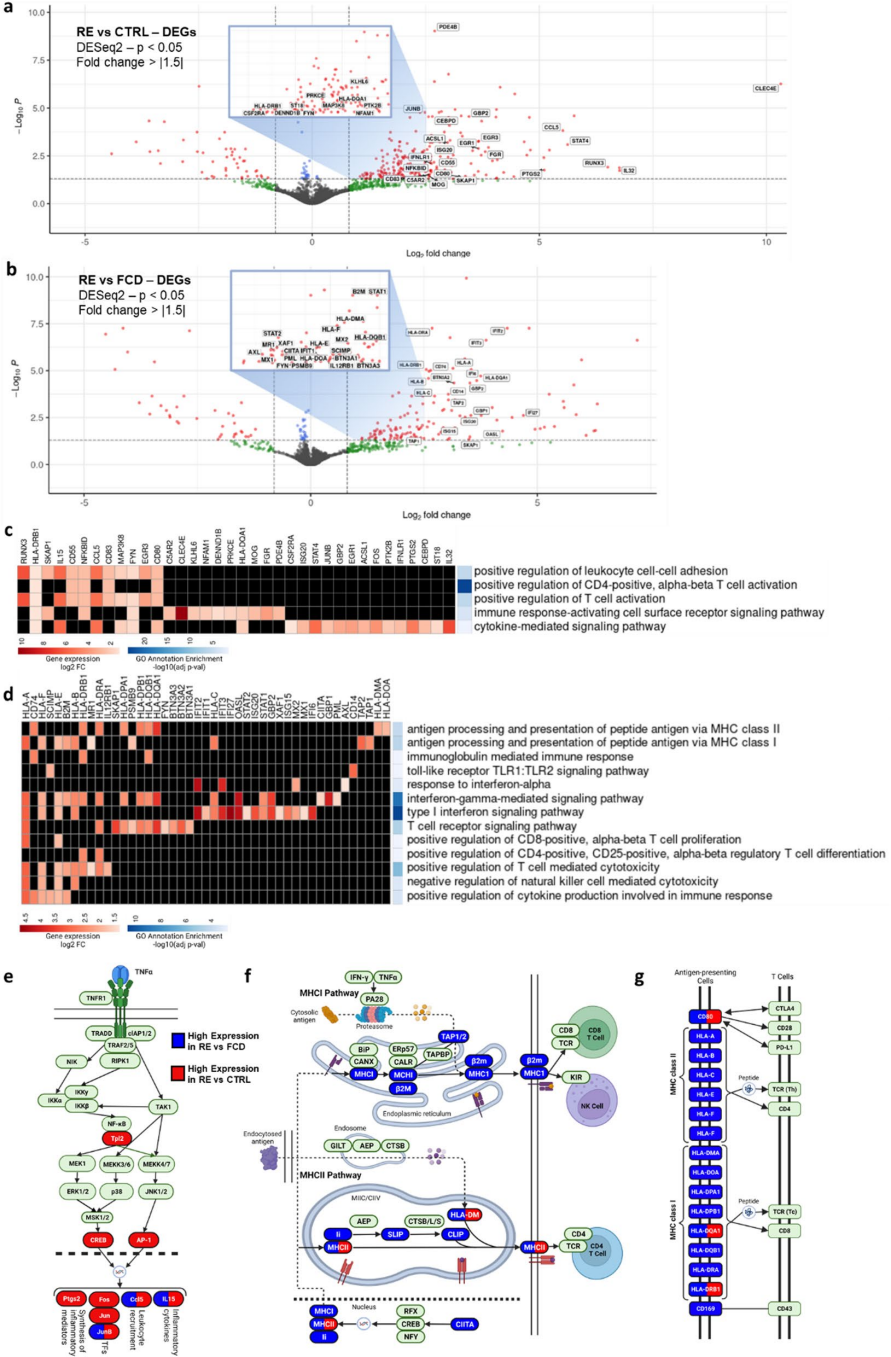
Gene expression signatures of microglia in Rasmussen encephalitis. **a** Volcano plot showing differentially expressed genes in RE vs CTRL (DESeq2 adjusted *p*-value < 0.05, FC > 1.5). Labeled genes are shown on heatmap in Fig. 2c. **b** Volcano plot showing differentially expressed genes in RE vs FCD (DESeq2 adjusted *p*-value < 0.05, FC > 1.5). Labeled genes are shown on heatmap in Fig. 2d. **c** Heatmap showing individual gene contribution (via positive fold-change) to GO biological process annotation enrichment in RE vs CTRL. **d** Heatmap showing individual gene contribution (via positive fold-change) to GO biological process annotation enrichment in RE vs FCD. **e–g** Selections from KEGG Pathways: TNF signaling pathway **e**, Antigen processing and presentation **f** and Cell adhesion molecules **g** highlighting differentially high expression of genes in RE vs both CTRL and FCD controls

We next investigated disease-specific changes in microglial gene expression using a pseudobulk comparison of RE versus both CTRL and FCD tissues in DESeq2. Pseudobulk analysis has been recently identified as a robust approach to avoid pseudo-replication bias inherent in single-cell gene expression studies [55]. Compared to CTRL tissue, our analysis revealed a total of 288 DEGs (237 with higher expression in RE samples, 51 with lower expression in RE, with at least a ± 1.5-fold change and an FDR-adjusted *p* value ≤ 0.05) (Fig. 2a, Additional file 2: Table 1). Compared to FCD tissue, our analysis revealed a total of 174 DEGs (134 with higher expression in RE samples, 40 with lower expression in RE, with at least a ± 1.5-fold change and an FDR-adjusted *p* value ≤ 0.05) (Fig. 2b, Additional file 13: Table 2). There was an overlap of 52 DEGs (39 with higher expression in RE samples, 13 with lower expression in RE) (Additional file 2: Fig. 2a-b) between the two disease-specific comparisons. Genes more abundantly expressed in RE were queried for biological process Gene Ontology (GO) annotation enrichment. Both sets of DEGs showed enrichment for immune mediation, including pathways for T-cell activation and cytokine signaling. In particular, DEGs highly expressed in RE vs. FCD were enriched for multiple interferon-mediated pathways, MHCI/II antigen presentation, CD8 + /CD4 + T-cell activation and Toll-like receptor pathways (Fig. 2c, d).

Next, we investigated the role of DEGs in well-studied biological pathways. Using the Kyoto Encyclopedia of Genes and Genomes (KEGG) Database Mapper Search Tool [25], we showed that many RE-abundant DEGs, compared to both CTRL and FCD, are in canonical immune pathways. These pathways include the TNFα pathway, known to promote inflammatory cytokine production [66] (Fig. 2e), both MHCI and MHCII components of the antigen presentation pathway (Fig. 2f) and the immune cell component of the cell adhesion pathway (Fig. 2g).

## Results

### Microglial heterogeneity in RE

**Fig. 3:**
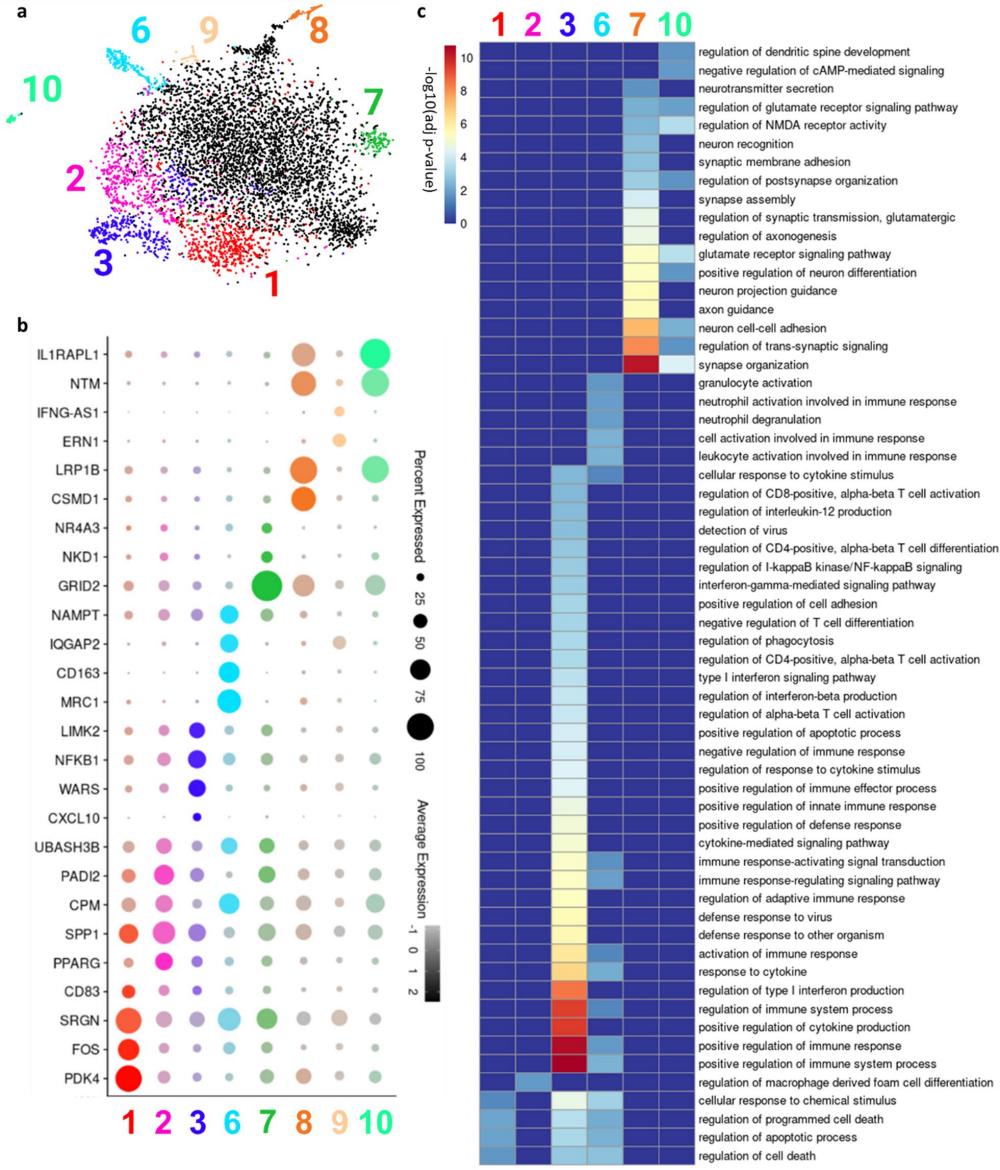
Microglial heterogeneity visualized via Louvain clustering. **a** tSNE plot showing eight clusters containing differentially high expression of genes with immune or inflammatory regulatory function. **b** Dot plot showing percent and average expression of a selection of differentially highly expressed genes in clusters from Fig. 3a with immune or inflammatory regulatory function **c** Heatmap showing enrichment of GO biological process annotations in clusters with enriched GO terms from Fig. 3a

To identify subpopulations of microglia that may be relevant to disease, we performed unbiased Louvain clustering on all microglia (Additional file 3: Fig. 3a) and determined the top differentially expressed genes for each subcluster (Additional file 3: Fig. 3b; Additional file 14: Table 3). Eleven subpopulations, or clusters, were identified. Note that each cluster, excluding cluster 7, contained cell populations from each disease condition (Additional file 4: Fig. 4a-c), though we determined that there was no statistically significant association between disease condition and microglial subpopulation (Additional file 4: Fig. 4d). Each cluster had expression of microglia-specific and homeostatic markers (Additional file 3: Fig. 3c). However, clusters 0 and 4 returned no significant DEGs and cluster 5 DEGs were nearly all ribosomal or mitochondrial genes. The remaining eight subpopulations had highly expressed gene markers involved in immune or inflammatory processes (Fig. 3ab) and were the focus of further analysis. Most of these eight clusters were defined by well-known pro-inflammatory markers. However, one population (Fig. 3b, cluster 6) was characterized by anti-inflammatory gene markers *CD163* and *MRC1*. Many of these markers, particularly in clusters 6,7,8 and 9, were more highly expressed in the RE microglia than the CTRL and FCD microglia (Additional file 5: Fig. 5).

DEGs in each cluster were queried for enriched biological process GO annotations. Six of the clusters were enriched for at least one GO term (Fig. 3c). Cluster 1 was enriched for apoptotic and cell death markers. Clusters 3 and 6 were enriched for regulation of immune processes including activation of immune cells (CD4 + /CD8 + T-cells, neutrophils) and inflammatory cytokine (*IL-12*, Type I interferon) production. Cluster 2 was enriched for regulation of macrophage derived foam cell differentiation, cells which have been implicated as pro-inflammatory in multiple neuroinflammatory disorders [68]. Clusters 7 and 10 were enriched for regulation of neuronal organization and signaling, including glutamate signaling pathways and glutamatergic synaptic transmission. Disruptions in homeostatic glutamate levels and signaling have been linked to excitotoxic damage from chronic seizures across a range of pathologies [8].

To examine the relationship between homeostatic microglia and immune-regulatory subclusters, we performed trajectory analysis. Clustered microglia were dimensionally reduced using Uniform Manifold Approximation and Projection (UMAP) (Additional file 6: Fig. 6a). A small group of cells in cluster 4, where homeostatic marker TMEM119 expression was highest, was chosen as the root node, representing pseudotime = 0 (Additional file 6: Fig. 6b). Gene expression pseudotime trajectories were calculated, and microglia were computationally ordered along the trajectories (Additional file 6: Fig. 6c). Nearly all trajectories went from cluster 4 through cluster 0 to a terminal cluster, excluding one trajectory leading directly to cluster 10. There is also a mid-to-late pseudotime trajectory leading through clusters as follows: 0,3,2,1, with cells in cluster 1 having some of the latest pseudotime scores, along with cluster 6 (Additional file 6: Fig. 6d). Spatial autocorrelation analysis revealed 4853 pseudotime differentially expressed genes (PDEGs) over the trajectories (Additional file 15: Table 4; Top 100). Visualization of the top 20 PDEGs (Moran’s I score) reveals that the PDEGs are most highly expressed in clusters 2,8,6 and 10 with many having high co-expression in clusters 8 and 10 (Additional file 7: Fig. 7). Furthermore, five of the top 20 PDEG’s were also regulators of immune response or inflammation highlighted in Fig. 3b.

### Spatial proteomic analysis of microglial nodules

**Fig. 4:**
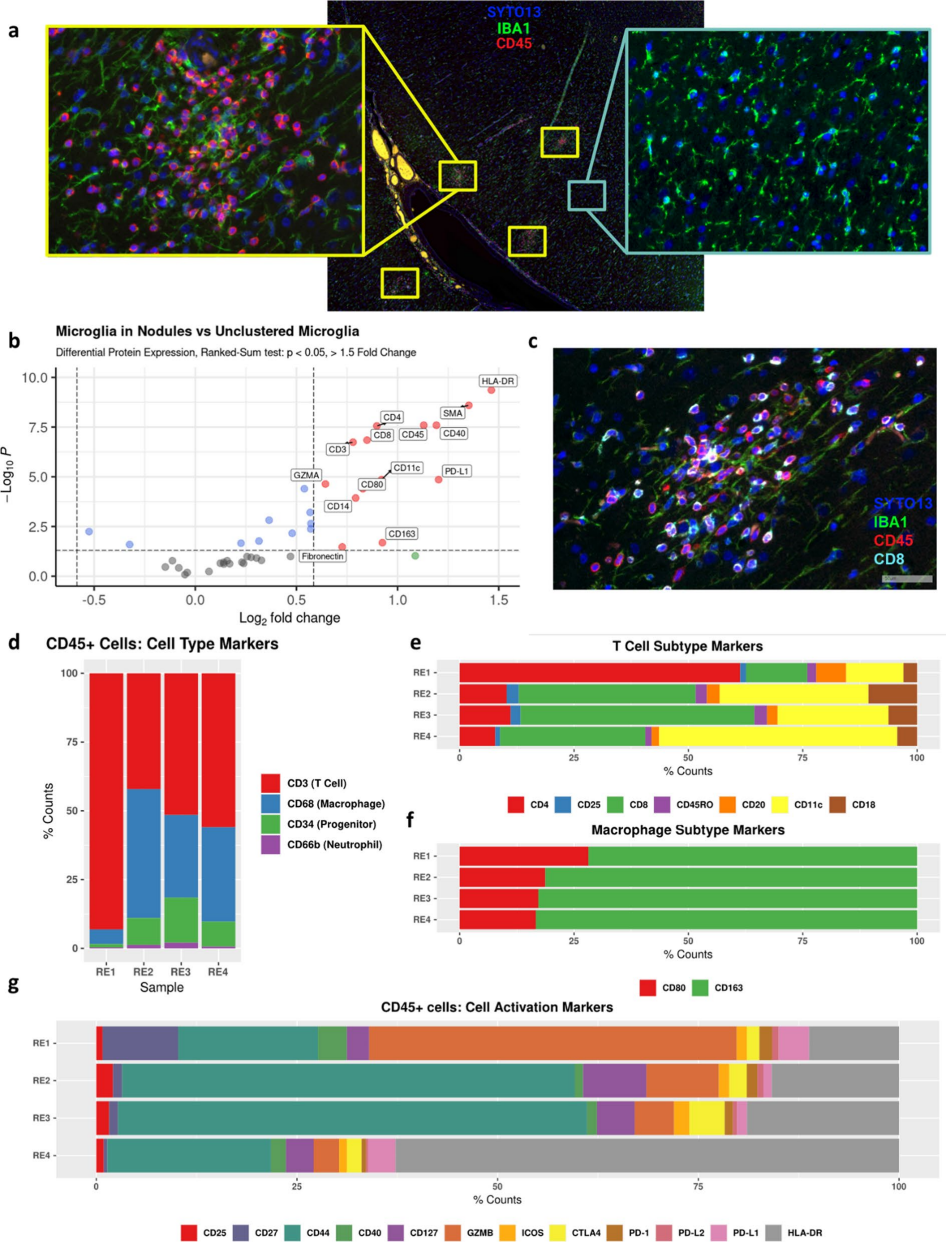
Microglia have distinct expression signatures based on spatial context in Rasmussen encephalitis. **a** Example ROIs representing a microglial nodule (left, yellow) versus unaggregated microglia (right, light blue) with immunofluorescent staining of microglia (IBA1, green), lymphocytes (CD45, red), and nuclei (SYTO13, blue). **b** Volcano plot of significant abundantly expressed proteins detected in microglial nodules versus unaggregated microglia. **c** 20 × micropictograph confirming high expression of CD8 in CD45+ cells near microglial nodules. **d-g** Relative cell type marker expression **d** T-cell subtype marker expression **e** macrophage subtype marker expression **f** cell activation markers **g** in CD45+ ROIs for each patient

To further profile RE microglia within their pathological context, we performed digital spatial protein profiling of brain tissue from patients using the Nanostring GeoMx platform (Additional file 8: Fig. 8a). The formation of microglial nodules in patient tissue has been reported as a key precedent to cytotoxic T-cell infiltration in RE [60]. We fluorescently labeled slides with IBA1 and CD45 to identify microglia and infiltrating lymphocytes, respectively, and compared ROIs containing microglial nodules versus unaggregated microglia within the same tissue section (Fig. 4a, Additional file 8: Fig. 8b). We quantified expression of 42 protein targets from the IBA1-positive fraction of each ROI (Additional file 9: Fig. 9) and performed differential expression analysis, which identified 14 highly differentially expressed proteins (DEPs) in microglia located in the nodules. Many of these proteins are known to be implicated in microglial activation (e.g., HLA-DR, CD80, CD40) or T-cell activity (e.g., CD8, GZMA; Fig. 4b). Indeed, immunofluorescent co-labeling of IBA1, CD45, and CD8 showed colocalization of CD8 on CD45 + lymphocytes in close proximity to microglial nodules (Fig. 4c). To further characterize invading immune cells localized within microglial nodules, we quantified the proportion of cell-type and cell-activation markers expressed within the CD45 + segment of the ROIs (Fig. 4d-g; Additional file 10: Fig. 10). T-cell markers dominated this signature in RE, with all four samples having evidence of CD3, CD8, CD4 expression (Fig. 4d–e). Concordantly, prominent T-cell activation markers, CD44 and HLA-DR, were highly represented in all four patients as well (Fig. 4g).

To connect spatial protein profiling to the single cell transcriptome, we analyzed a selection of six genes that encode for DEPs abundantly expressed in microglial nodules (Additional file 11: Fig. 11a). In an effort to locate microglia from a nodule in the snRNA-Seq microglia subset, the expression density of each DEP gene was plotted in the TSNE projection of the microglia (Additional file 11: Fig. 11b). A combined joint density plot revealed that some microglia in cluster 3 express all six genes that encode the microglial nodule DEPs (Additional file 11: Fig. 11c).

## Discussion

In this study we applied single-nucleus transcriptomic and spatial proteomic approaches to the study of Rasmussen encephalitis resected brain tissue. Our results confirm and extend prior observations of immune-mediated pathogenesis in RE. First, we showed an expanded population of microglia and infiltrating lymphocytes in RE patients compared to brain tissue from both epilepsy-only controls and unaffected individuals. By examining the transcriptomes of microglial subclusters, we were able to identify extensive heterogeneity representing microglia in various states of activation. Finally, we were able to place that observation in pathological context by comparing expression profiles of microglia physically located within microglial nodules versus unaggregated. Microglia located in nodules expressed classic activation markers and were closely located to CD8+ T-cells.

Efforts to understand the immune-mediated pathogenicity of RE have largely focused on the study of infiltrating T-cell populations [1, 42, 52]. While microglia-associated inflammation has been studied across several different pathologies [19, 37, 54], few studies have analyzed microglial mechanisms in RE [11, 60]. Troscher et al. were the first to analyze microglial nodules in the context of RE. Our snRNA-seq differential expression analysis of RE microglia aligns with and expands on the bulk microarray gene set enrichment analysis (GSEA) of stage I and II nodules [60]. The RE microglia overexpress genes involved in MHC-I and MHC-II antigen presentation, and cytokine signaling, particularly of the interferon family. Furthermore, our data show that RE microglia have significantly higher expression of genes involved in positive regulation of specific T-cell activation, differentiation and cytotoxicity. Also, our data show support for the hypothesis that Toll-like receptors are activated upstream of immune pathways. The TLR1:TLR2 signaling pathway GO annotation is enriched in RE microglia, known to have downstream activating effects on the TNFα pathway [40]. Interestingly, in microglia, TNFα has been shown to be a potent stimulus of TLR2 expression [57], potentially creating a feedback loop for immune activation.

Performing differential expression analysis on microglial subclusters allowed us to identify and quantify the heterogeneity of microglial function in the context of RE. Looking at the most differentially expressed immune markers in each cluster revealed populations of microglia with varied inflammation profiles. Not surprisingly, there is a subset of microglia (cluster 3) with differentially high gene expression representing a pro-inflammatory Th1 immune response (*NF-κB, CXCL10*) [43, 67] and innate inflammatory responses through TLR2/4 production (*WARS1*) [38]. These cells also had high co-expression of *LIMK2*, a known biomarker for necrotic neuronal death during epileptic seizure [28]. Interestingly, a different microglial population (cluster 6) has a more ambiguous role in inflammation. Having high expression of anti-inflammatory markers *CD163* and *CD206* (*MRC1*)) [30, 50], these microglia also co-express pro-inflammatory *NAMPT* and *IQGAP2*, a ligand for, and promotor of *TLR4*, respectively [6, 20]. The dichotomy of these populations suggests a more spatially nuanced, dynamic inflammasome and implicates variation in Toll-like receptor signaling as potential mediator, reinforcing previous observations made by Luan et al. [34]. It is important to note that while microglia from all microglial subclusters were represented in each disease condition, there was an overall increased proportion of microglia in RE compared to the other conditions (Additional file 4: Fig. 4b-c). However, when analyzing expression of genes that regulate immune function or inflammation, particularly those highlighted in Fig. 3b, many are more abundantly expressed in RE microglia than in FCD or CTRL populations. Furthermore, our proteomic data demonstrate that the spatial organization of these microglial subpopulations is altered in the context of RE.

GO annotation enrichment analysis of microglia cluster DE genes revealed additional regulated biological processes beyond inflammation. Microglia in cluster 2 overexpress genes that regulate macrophage-derived foam cell differentiation, indicating that the microglia contain cellular debris, presumably from neurodegeneration. Although foamy microglia are common in multiple sclerosis (MS), another neurological disease due to immune-mediated pathogenesis [21, 68], these cells have not been analyzed in the context of RE. These cells could provide crucial spatiotemporal information about the neurodegenerative microenvironment of RE.

Other microglial subpopulations have similarities to microglial phenotypes studied in Alzheimer Disease (AD). Cluster 5 has abundantly high expression of FTL and FTH1, genes that encode for ferritin subunits. A similar iron-loading microglial phenotype, described by Kenkhuis et al., has been found in AD patients with high *β*-amyloid, high tau pathologies [27]. Two different clusters, 7 and 10, abundantly expressed genes such as *GRID2,* related to microglia-neuron crosstalk and the organization of synapse and postsynaptic organization. It is well documented that a major role of microglia is to regulate synaptic development and pruning, and dysregulation of these processes leads to inflammatory and epileptogenic disorders [4, 5]. Gerrits et al. described a similar microglial phenotype in the context of AD, particularly in tissue with tau pathology. These *GRID2* + microglia were also enriched for gene ontology annotations related to neurotrophic functions like synapse organization and axonogenesis [19]. Understanding the role of these phenotypically related microglia across neurodegenerative diseases could offer insight into how microglia respond to neuroinflammatory pathologies. Microglia from cluster 7 and 10 also express genes that regulate *NMDA* receptor activity and are responsible for microglial process extension toward neurons during seizure, induced by increased global glutamate levels [16]. Additionally, these microglia abundantly express genes that regulate glutamate receptor signaling pathways and glutamatergic synaptic transmission. Several studies have shown that irregular glutamate levels and aberrant glutamatergic signaling lead to inflammation and epilepsy [8, 14]. Analysis of these microglia could offer insight into the transcriptomic landscape of epileptogenic microglia not just for RE, but for other disorders.

Pseudotime trajectory analysis of snRNA-Seq data suggests that while some microglial subpopulations have unique paths of differentiation from a homeostatic state, others may be temporally related along one or more differentiation trajectories. All trajectories from homeostasis to activated microglia, except one, travel through a common microglial population, cluster 0. All the microglia in cluster 0 have early to mid pseudotime scores and cluster 0 has no differentially high expression of genes. This suggests that the microglia in this cluster represent an initial differentiation state in which expression of homeostatic markers drops but inflammatory markers are not yet defined. In contrast, there are trajectories through multiple populations of inflammatory microglia, suggesting a temporal relationship mediated by cell signaling. A trajectory through clusters 3,2,1 emphasizes the role of both *NFKB1*, *CXCL10* and *WARS* as initiators of proinflammatory signaling, and their potential to affect function of nearby microglia [13, 32, 38]. Spatial autocorrelation in Monocle3 revealed PDEGs that had appreciable overlap with DEGs generated in Seurat. Interestingly, of the PDEGs with a top 20 Moran’s I scores (Additional file 7: Fig. 7), five were DEGs highlighted in Fig. 3b as markers of immune regulation and inflammation. This correlation reinforces that in RE-affected microglia, the dominant drivers of microglial activation and deviation from homeostasis are modulators of immune response and inflammation. The top five PDEGs have differentially high expression in both clusters 8 and 10, however the expression occurs at different pseudotimes and the clusters are along different trajectories, indicating that functionally similar populations of microglia may have different activating drivers. Understanding the mechanisms of activation for disease-specific microglia across pseudotime trajectories can lead to novel targets for immune checkpoint therapeutics for neuroinflammatory disease.

While snRNA-seq provides transcriptome-wide data at single-cell resolution, it lacks spatial context and only infers in vivo protein abundance. Having high-plex, spatially-resolved proteomic data allowed us to further elaborate on the immunohistological data of Troscher et al. in two ways. First, we analyzed the differentially expressed proteins (DEPs) in microglial nodules. Second, we quantified the immune proteins of infiltrating CD45 + lymphocytes at microglial nodule locations. Microglial nodules consisted exclusively of IBA1 + cells with an activated, ameboid phenotype. Non-clustered microglia regions were selected to maximize homeostatic, ramified phenotypes. Not surprisingly, differential protein expression analysis revealed that HLA-DR, a classic marker of immune activation [56, 64], was expressed nearly threefold higher in clustered microglia versus non-clustered. Other proinflammatory markers were also highly expressed in the nodules. CD40 is a microglial TNF receptor that is important in T-cell activation, stimulated by IFN-*γ* through the TNF*α* pathway, and has been implicated in other autoimmune disorders [12, 39]. Furthermore, when CD40 is bound to T-cell ligand CD40L, it promotes an increase in expression of CD80, another proinflammatory costimulatory molecule [65]. Alternatively, we see high relative expression of CD163 and MRC1, anti-inflammatory markers. Other DEPs have a more elusive role in the immune microenvironment of the nodules. PD-L1 + microglia have been shown to, in most cases, correlate with inhibition of CD8 + T-cells and reduce inflammation, although the mechanism of protection remains unclear [35]. Similarly, CD11c + microglia have protective and regenerative effects in neuroinflammatory conditions [9]. CD14 has been shown to be an essential regulator of TLR4-mediated damage and disease response of microglia [24]. Our proteomic analysis provides spatial context for the dynamic mix of inflammatory signaling introduced by our single-nucleus transcriptomic analysis. Mapping the expression of genes that encode for nodule-enriched DEPs onto the microglia tSNE projection reveals that microglia in cluster 3 are most likely to be from a microglial nodule. This logically correlates with neuroinflammatory function of microglial nodules. Not only does cluster 3 have differentially high expression of *NFKB1*, *CXCL10, LIMK2* and *WARS*, all contributors to pro-inflammatory signaling [3, 13, 32, 38], but also the highest enrichment of GO annotations for positive regulation of immune response and cytokine, including type I interferon, production.

Immune targets for proteomic analysis of invading CD45+ lymphocytes were split into two groups, those that traditionally designate immune cell types and subtypes (Fig. 4d–f) and those that designate immune activation state (Fig. 4g). Although T-cell markers dominate the signal for CD45 + invading immune cells, there is evidence that invading monocyte-derived macrophages are present near the microglial nodules (Fig. 4d, f). Using the relative abundances of T-cell subtype and activation markers allows us to determine shifts in T-cell populations across samples. In RE4, based on CD3-CD8-CD4-CD11c expression, some of the lymphocytes are effector T-cells also known as cytotoxic T-cells (CTLs) [31] and others are likely a subset of regulatory T-cells (Tregs) shown to suppress CD4+ T-cells [49, 63]. RE2 and RE3 have similar protein expression profiles to RE4, however the lower CD11c expression and higher CD44 and suggest that the CTL:Treg ratio of present lymphocytes is higher [47]. In RE1 however, we observed much lower CD8 and CD44 expression, suggesting a lack of CTLs. Instead, we observed expanded expression of CD3, CD4 and GZMB. CD4+ GZMB+ T-cells have been shown to be a hallmark of several systemic autoimmune diseases and are associated with inflammation and tissue damage [45]. In addition to their helper and cytotoxic functions, these CD4+ T-cells are hypothesized to promote CD8+ T-cell effector processes via secreted cytokines [1]. Our analysis highlights the heterogeneity of patient immune response to RE and could help explain why immunotherapy has been largely ineffective in controlling RE progression [58].

## Conclusions

These findings highlight the important role of microglia in the pathogenesis of Rasmussen encephalitis and confirm the significance of microglial nodules in promoting cytotoxic T-cell activity. Our work further underscores the advantages of technology, leveraging single-cell and spatial resolution for cell-type and location specific contributions to RE pathology. By performing multiomic analysis of disease affected microglia, we gain a deeper understanding of aberrant intra- and inter-cellular signaling leading to the progression of inflammation and neurotoxicity of RE. Our work ultimately demonstrates new and powerful methodologies that may guide future research toward elucidating the origin of RE pathogenesis and effective therapeutic intervention.

## Supplementary Information

**Additional file 1**. QC and cell typing for snRNA-seq data. **a**, **b** tSNE plots showing raw RNA and UMI counts per nucleus. **c**, **d** tSNE plots showing SCT-normalized RNA and UMI counts per nucleus. **e** tSNE plot showing mitochondrial transcript expression as a percent of total expression per nucleus. **f** Heatmap showing expression of common marker genes for annotated cell type. **g**, **h** Stacked bar graphs showing relative cell type per disease condition (**g**) and sample (**h**).

**Additional file 2.** Overlap of DEGs in RE relative to FCD and CTRL. a,b Venn diagrams showing the number of overlapping genes expressed differentially high (**a**) and differentially low (**b**) in RE vs CTRL and FCD. **c** Four-way plot showing FCD expression relative to RE vs CTRL expression relative to RE for each gene.

**Additional file 3.** Microglial markers and DEGs in microglial Louvain clusters. **a** tSNE plot of microglia showing all eleven Louvain clusters. **b** Violin plots of top five most abundant differentially expressed genes in each microglial cluster, if applicable. **c** Violin plots illustrating the per-cluster expression of microglia-specific and homeostatic microglial markers.

**Additional file 4.** Sample and disease condition contribution to microglial clusters. **a** Side-by-side tSNE plot of microglia with accompanying stacked bar graph showing the percent of cells in cluster by sample. **b** tSNE plot of microglia in which each cell is colored by disease condition. **c** Stacked bar graph showing the percent of cells in disease condition by microglial cluster. **d** Box plot showing the distribution of cell type diversity statistics in each disease condition.

**Additional file 5.** Immune modulation and inflammation marker expression differs by disease condition. Ridge plots showing the expression of immune and inflammation markers highlighted in Figure 3, separated by disease condition with accompanying tSNE of microglia for reference.

**Additional file 6.** Pseudotime trajectory analysis. **a** UMAP plot of microglia showing all eleven Louvain clusters. **b** UMAP density plot showing the density of TMEM119 expression. Red star indicates root node for cell ordering. **c** UMAP plot showing pseudotime trajectories, where cells are colored by pseudotime score. Red star indicates root node for cell ordering. **d** Ridge plot displaying the pseudotime score of cells in each microglia cluster.

**Additional file 7.** Top PDEGs highlight diversity of pseudotime trajectories. Scatter plots of cells showing expression of top 20 PDEGs (by Moran’s I score) vs pseudotime where cells are colored by microglial cluster. Red stars indicate gene is also an immune or inflammatory marker highlighted in Figure 3b.

**Additional file 8.** Nanostring GeoMx workflow and ROI segmenting. **a** Schematic detailing the workflow of the GeoMx digital spatial profiler platform. **b** 20x micropictographs displaying single channel CD45 and IBA1 images, the combined image, and how a typical ROI containing a microglial nodule was segmented by intensity thresholding.

**Additional file 9.** IBA1+ ROI segment protein expression. Heatmap showing protein expression of 42 protein targets in each IBA1+ ROI segment for each sample.

**Additional file 10.** CD45+ ROI segment protein expression. Heatmap showing protein expression of 42 protein targets in each CD45+ ROI segment for each sample.

**Additional file 11.** DEP gene expression in microglia snRNA-Seq. **a** Volcano plot of significant abundantly expressed proteins detected in microglial nodules versus unaggregated microglia. Red shape and labels highlight DEPs selected for single-nucleus microglia analysis. **b** tSNE density plots displaying gene expression density in microglia of markers selected in **a**. **c** tSNE density plot showing the expression density in microglia where all selected markers are expressed.

**Additional file 12: Table S1**. Differentially Expressed Genes in Microglia, RE vs CTRL.

**Additional file 13: Table S2**. Differentially Expressed Genes in Microglia, RE vs FCD.

**Additional file 14: Table S3**. Top Highly Differentially Expressed Genes in Microglia Subpopulations.

**Additional file15: Table S4**. Top 100 Differentially Expressed Genes Across Pseudotime Trajectories.

## Data Availability

The datasets generated from this study are available from the corresponding author upon reasonable request.

## Abbreviations

RE: Rasmussen encephalitis
AD: Alzheimer disease
MS: Multiple sclerosis
snRNA-Seq: Single nucleus RNA sequencing
FCD: Focal cortical dysplasia
CTRL: Unaffected autopsy control
CTDS: Cell type diversity statistic
DEG: Differentially expressed gene
DEP: Differentially expressed protein
PDEG: Pseudotime differentially expressed gene
FFPE: Formalin -fixed, paraffin embedded
PBS: Phosphate buffered saline
FACS: Fluorescence-activated cell sorting
PCA: Principal component analysis
tSNE: T-distributed stochastic neighbor embedding
UMAP: Uniform manifold approximation and projection
MAST: Model-based analysis of single-cell transcriptomics
GO: Gene ontology
KEGG: Kyoto Encyclopedia of genes and genomes
FDR: False discovery rate
ROI: Region of interest
