# Supplementary figures and images for: Molecular and spatial heterogeneity of microglia in Rasmussen encephalitis
Source: Acta Neuropathol Commun. 2022 Nov 21;10:168. doi: 10.1186/s40478-022-01472-y (PMC9677917; doi:10.1186/s40478-022-01472-y)

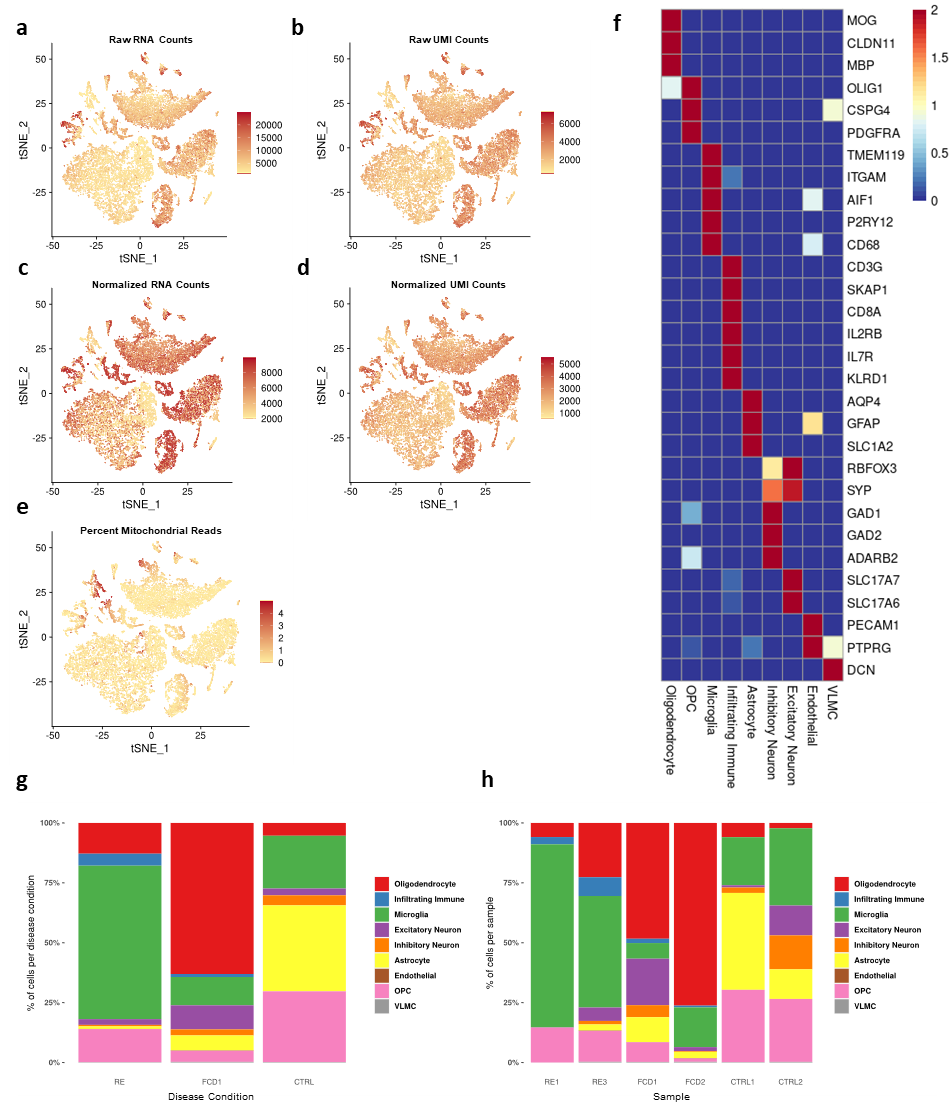

Supplement: Supplementary file 1 — Additional file 1. QC and cell typing for snRNA-seq data. a, b tSNE plots showing raw RNA and UMI counts per nucleus. c, d tSNE plots showing SCT-normalized RNA and UMI counts per nucleus. e tSNE plot showing mitochondrial transcript expression as a percent of total expression per nucleus. f Heatmap showing expression of common marker genes for annotated cell type. g, h Stacked bar graphs showing relative cell type per disease condition (g) and sample (h). [file 40478_2022_1472_MOESM1_ESM.docx]

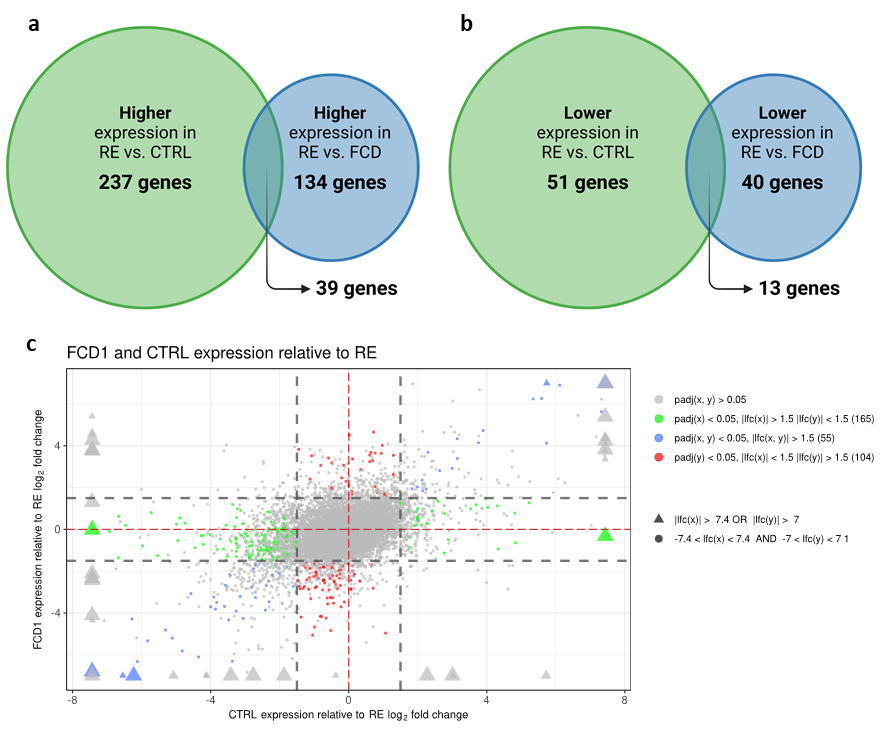

Supplement: Supplementary file 2 — Additional file 2. Overlap of DEGs in RE relative to FCD and CTRL. a,b Venn diagrams showing the number of overlapping genes expressed differentially high (a) and differentially low (b) in RE vs CTRL and FCD. c Four-way plot showing FCD expression relative to RE vs CTRL expression relative to RE for each gene. [file 40478_2022_1472_MOESM2_ESM.docx]

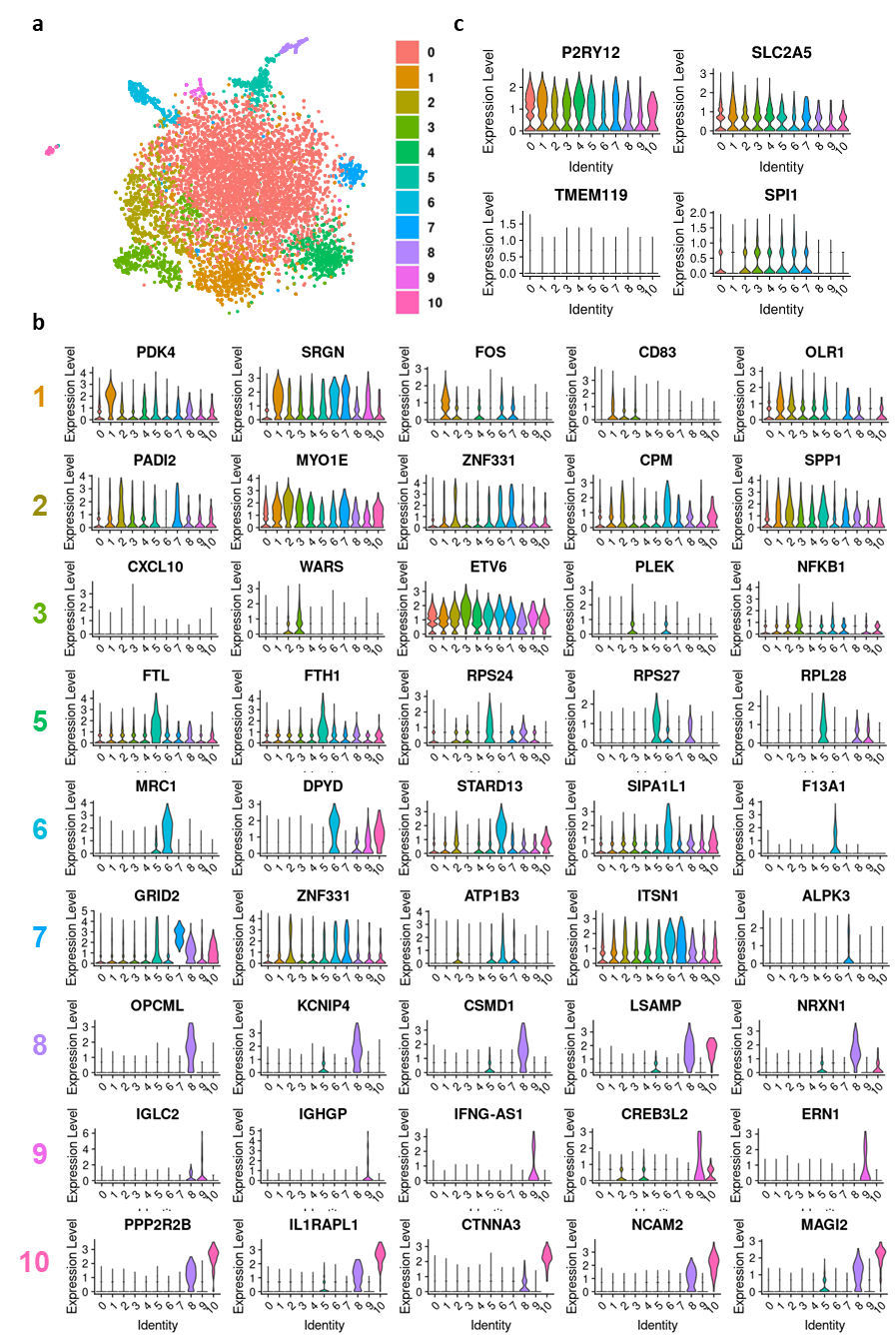

Supplement: Supplementary file 3 — Additional file 3. Microglial markers and DEGs in microglial Louvain clusters. a tSNE plot of microglia showing all eleven Louvain clusters. b Violin plots of top five most abundant differentially expressed genes in each microglial cluster, if applicable. c Violin plots illustrating the per-cluster expression of microglia-specific and homeostatic microglial markers. [file 40478_2022_1472_MOESM3_ESM.docx]

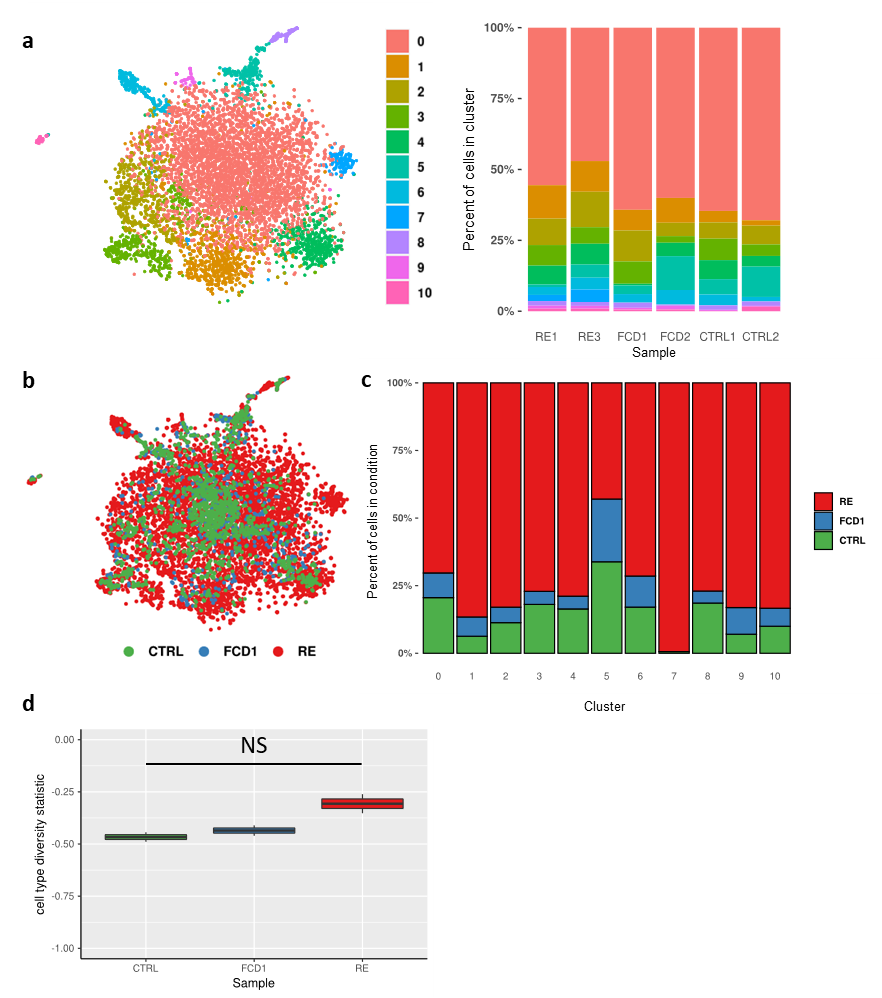

Supplement: Supplementary file 4 — Additional file 4. Sample and disease condition contribution to microglial clusters. a Side-by-side tSNE plot of microglia with accompanying stacked bar graph showing the percent of cells in cluster by sample. b tSNE plot of microglia in which each cell is colored by disease condition. c Stacked bar graph showing the percent of cells in disease condition by microglial cluster. d Box plot showing the distribution of cell type diversity statistics in each disease condition. [file 40478_2022_1472_MOESM4_ESM.docx]

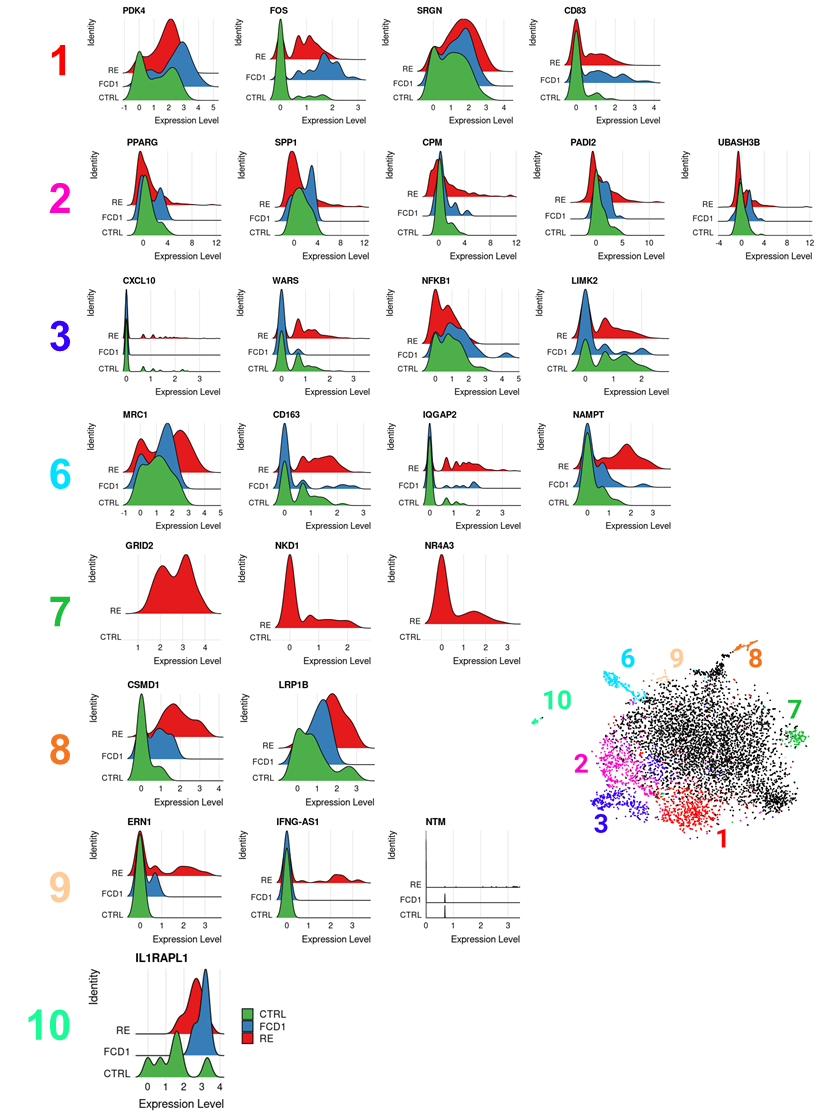

Supplement: Supplementary file 5 — Additional file 5. Immune modulation and inflammation marker expression differs by disease condition. Ridge plots showing the expression of immune and inflammation markers highlighted in Figure 3, separated by disease condition with accompanying tSNE of microglia for reference. [file 40478_2022_1472_MOESM5_ESM.docx]

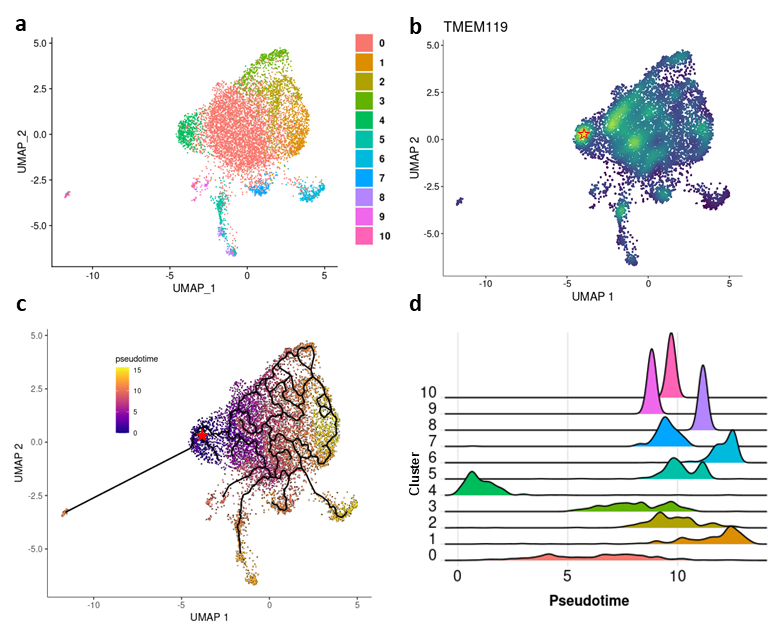

Supplement: Supplementary file 6 — Additional file 6. Pseudotime trajectory analysis. a UMAP plot of microglia showing all eleven Louvain clusters. b UMAP density plot showing the density of TMEM119 expression. Red star indicates root node for cell ordering. c UMAP plot showing pseudotime trajectories, where cells are colored by pseudotime score. Red star indicates root node for cell ordering. d Ridge plot displaying the pseudotime score of cells in each microglia cluster. [file 40478_2022_1472_MOESM6_ESM.docx]

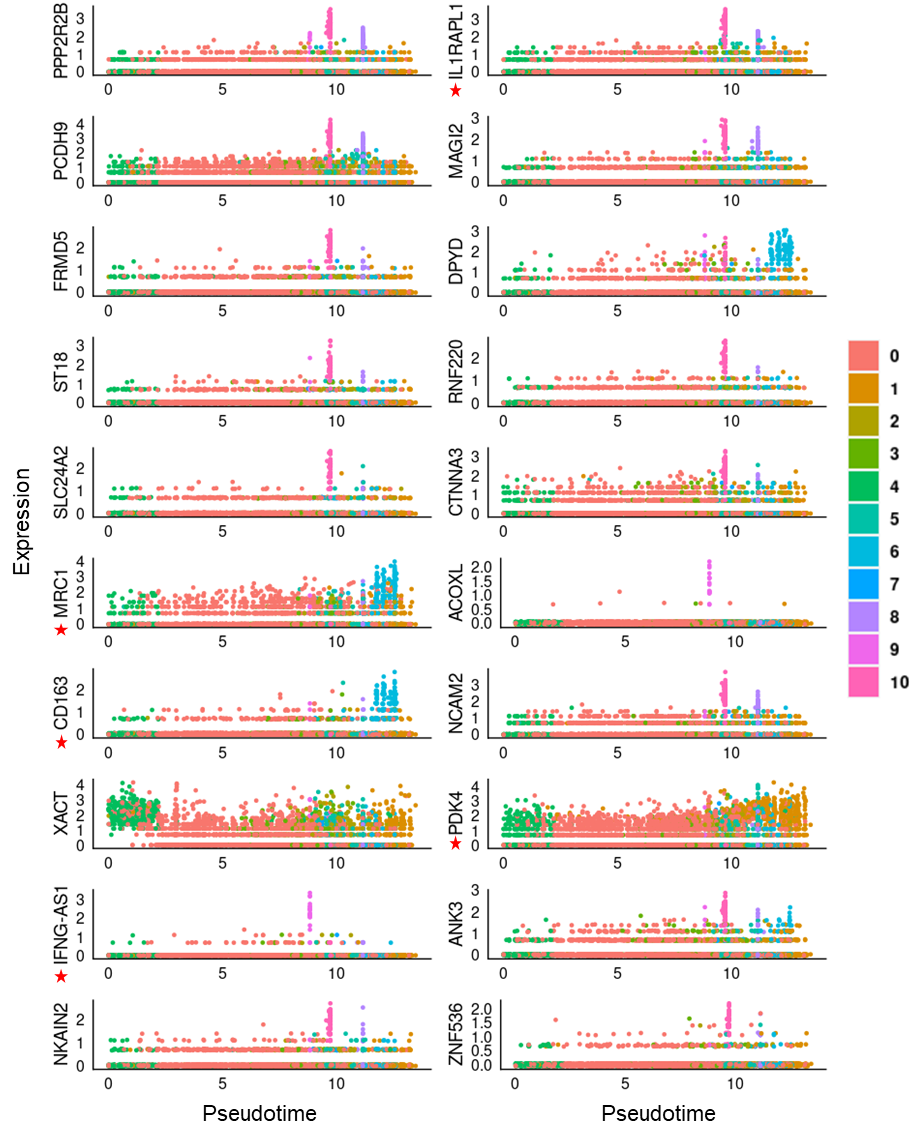

Supplement: Supplementary file 7 — Additional file 7. Top PDEGs highlight diversity of pseudotime trajectories. Scatter plots of cells showing expression of top 20 PDEGs (by Moran’s I score) vs pseudotime where cells are colored by microglial cluster. Red stars indicate gene is also an immune or inflammatory marker highlighted in Figure 3b. [file 40478_2022_1472_MOESM7_ESM.docx]

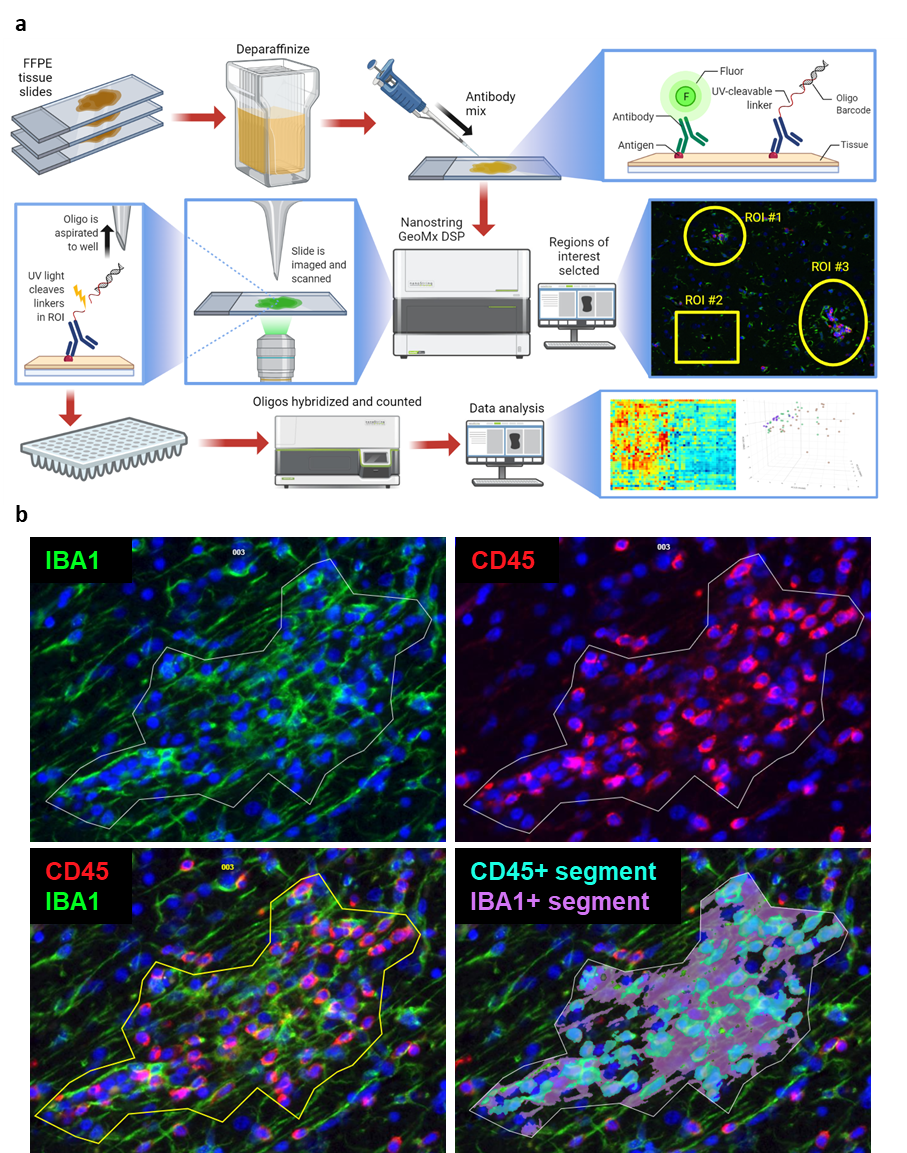

Supplement: Supplementary file 8 — Additional file 8. Nanostring GeoMx workflow and ROI segmenting. a Schematic detailing the workflow of the GeoMx digital spatial profiler platform. b 20x micropictographs displaying single channel CD45 and IBA1 images, the combined image, and how a typical ROI containing a microglial nodule was segmented by intensity thresholding. [file 40478_2022_1472_MOESM8_ESM.docx]

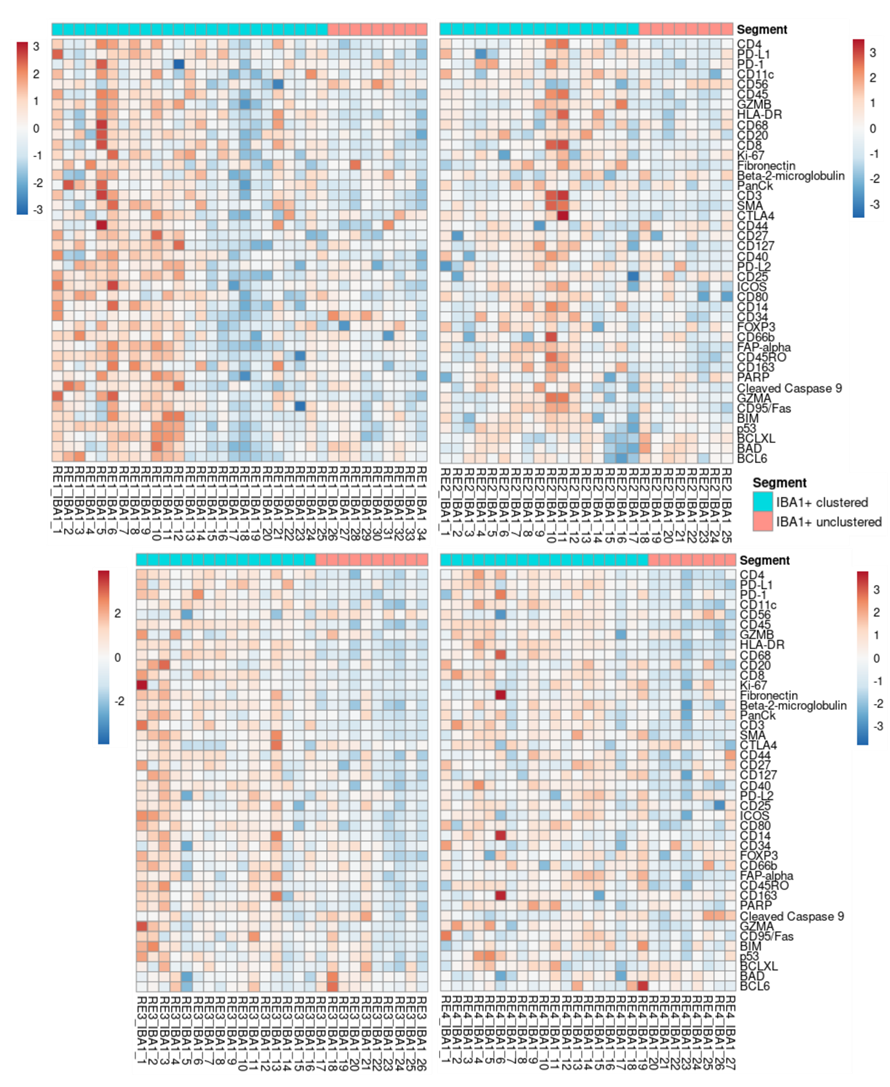

Supplement: Supplementary file 9 — Additional file 9. IBA1+ ROI segment protein expression. Heatmap showing protein expression of 42 protein targets in each IBA1+ ROI segment for each sample. [file 40478_2022_1472_MOESM9_ESM.docx]

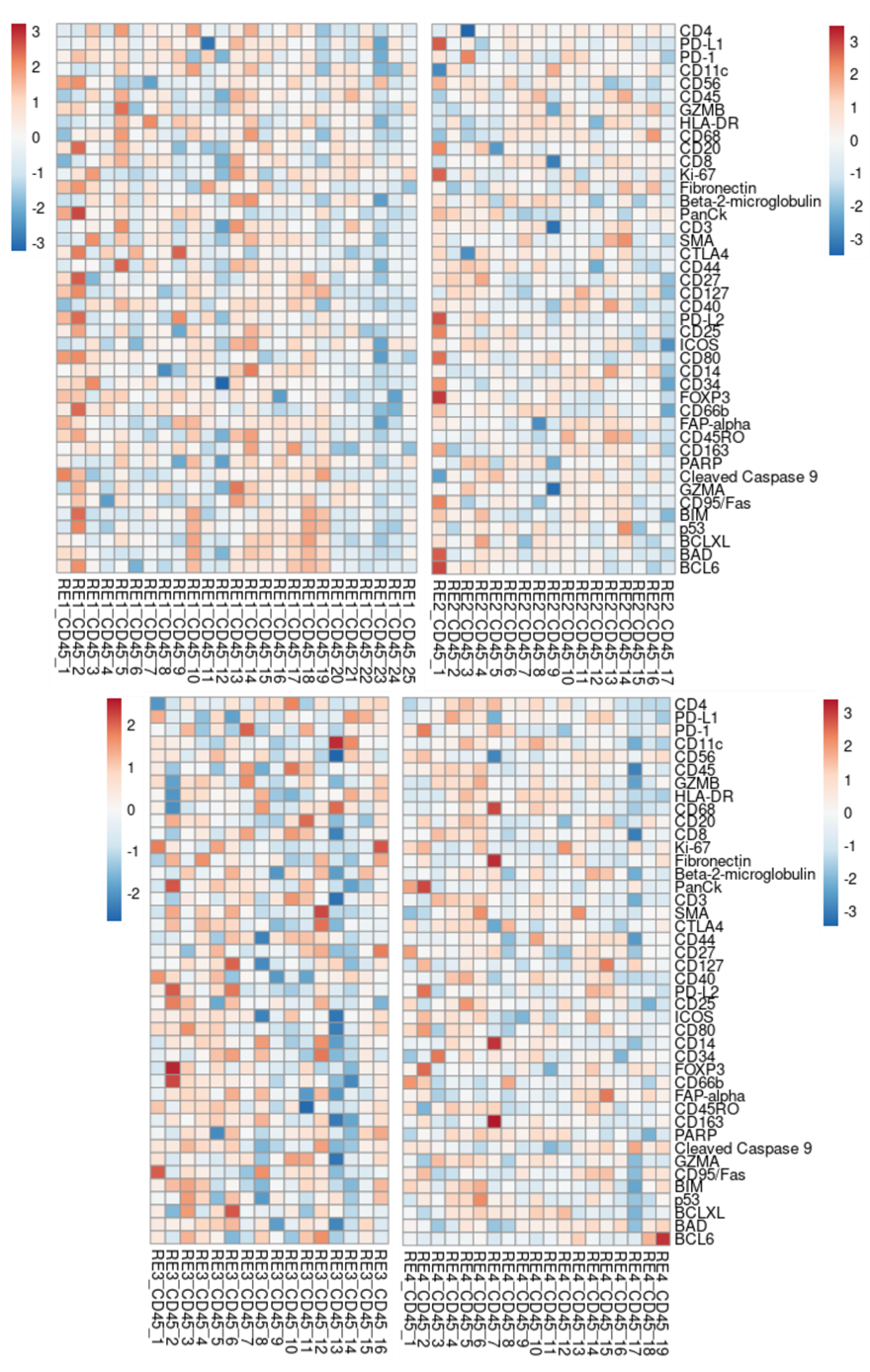

Supplement: Supplementary file 10 — Additional file 10. CD45+ ROI segment protein expression. Heatmap showing protein expression of 42 protein targets in each CD45+ ROI segment for each sample. [file 40478_2022_1472_MOESM10_ESM.docx]

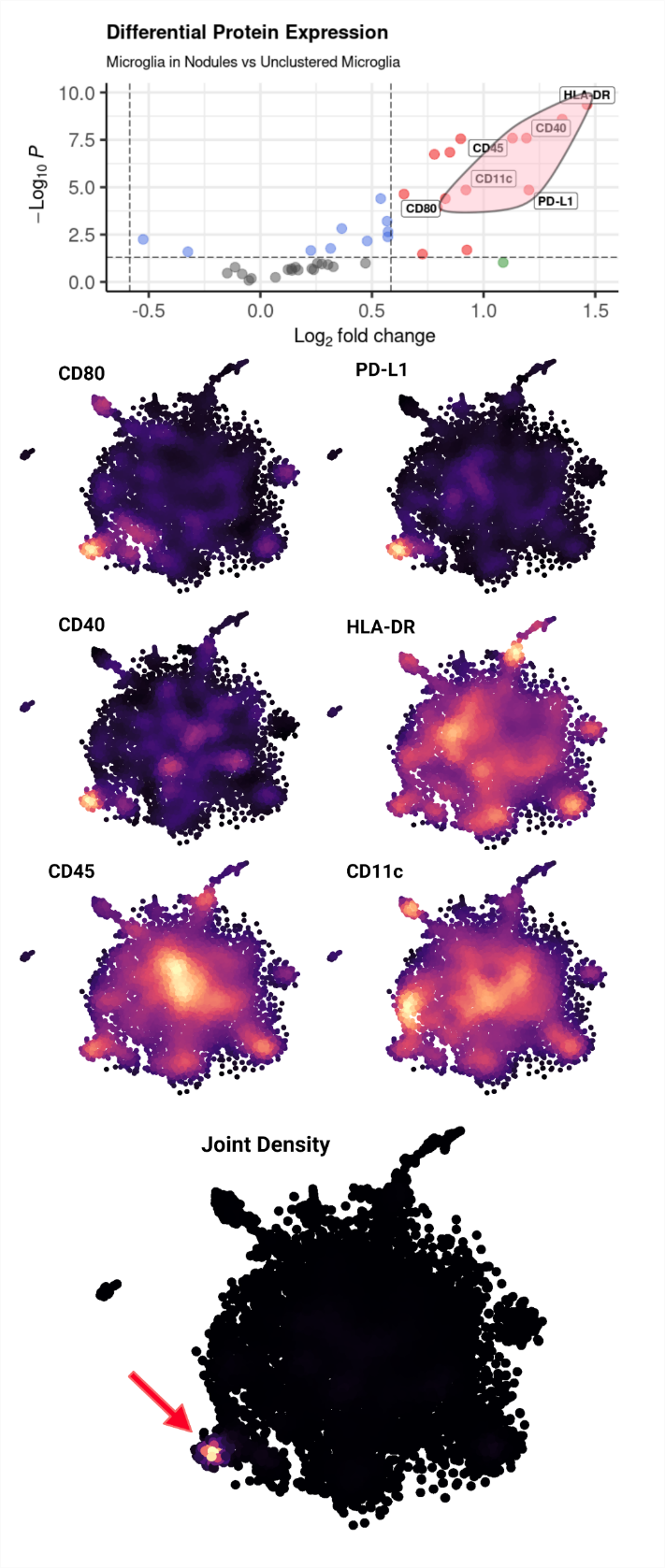

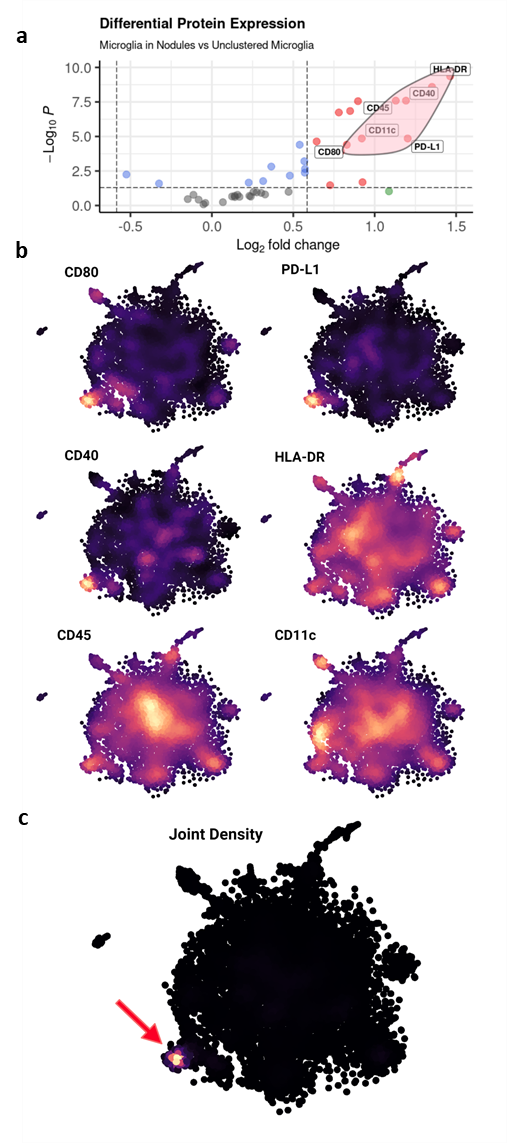


**b**

**a**

**c**

Supplement: Supplementary file 11 — Additional file 11. DEP gene expression in microglia snRNA-Seq. a Volcano plot of significant abundantly expressed proteins detected in microglial nodules versus unaggregated microglia. Red shape and labels highlight DEPs selected for single-nucleus microglia analysis. b tSNE density plots displaying gene expression density in microglia of markers selected in a. c tSNE density plot showing the expression density in microglia where all selected markers are expressed. [file 40478_2022_1472_MOESM11_ESM.docx]
